# Nanofibrous chiral supramolecular assembly-derived self-healing hydrogels with polyethylene glycol†

**DOI:** 10.1039/d4na00353e

**Published:** 2024-06-10

**Authors:** Makoto Takafuji, Kenji Kawamoto, Nanami Hano, Mako Otsuki, Hirotaka Ihara

**Affiliations:** a Faculty of Advanced Science and Technology, Kumamoto University 2-39-1 Kurokami, Chuo-ku Kumamoto 860-8555 Japan takafuji@kumamoto-u.ac.jp ihara@kumamoto-u.ac.jp +81-96-342-3661; b International Research Organization for Advanced Science and Technology (IROAST), Kumamoto University 2-39-1 Kurokami, Chuo-ku Kumamoto 860-8555 Japan

## Abstract

Unique polymer hydrogels with unusual cross-linking networks and self-healing properties have been recently reported. In this study, we fabricated hybrid hydrogels consisting of a chiral supramolecular one-dimensional assembly of glutamide-derived lipids bearing pyridinium head groups (*G*-Py^+^) cross-linked with termini-anionised hydrophilic polyethylene glycol polymers (S-PEG_*n*_-S). The cationic group-linked *G*-Py^+^ forms nanotubular aggregates in water. *G*-Py^+^/S-PEG_*n*_-S aqueous mixtures formed hydrogels at certain concentrations and ambient temperatures. The terminal anionic sulfate groups play a key role in hydrogel formation, as evidenced by the absence of gelation in *G*-Py^+^/PEG_*n*_. The negative circular dichroism signal observed for pyridinium exhibited a blue shift upon the addition of S-PEG_*n*_-S but maintained its signal intensity even with excess S-PEG_*n*_-S, suggesting the chiral orientation of the nanofibrous *G*-Py^+^ self-assembly preserved even complexation with S-PEG_*n*_-S in hydrogel. The hybrid hydrogel of sulfated polyethylene glycol with nanofibrous chiral supramolecular assembly exhibited self-healing property at a temperature below the gel-to-liquid crystalline phase transition (*T*_C_) of *G*-Py^+^ aggregates, which was evidenced by the inversion fluid method and viscoelastic measurements.

## Abstract 2

The hybrid hydrogel of nanofibrous chiral supramolecular assemblies with sulfated polyethylene glycol exhibited self-healing property at a temperature below the gel-to-liquid crystalline phase transition of chiral assemblies.

## Graphical abstract

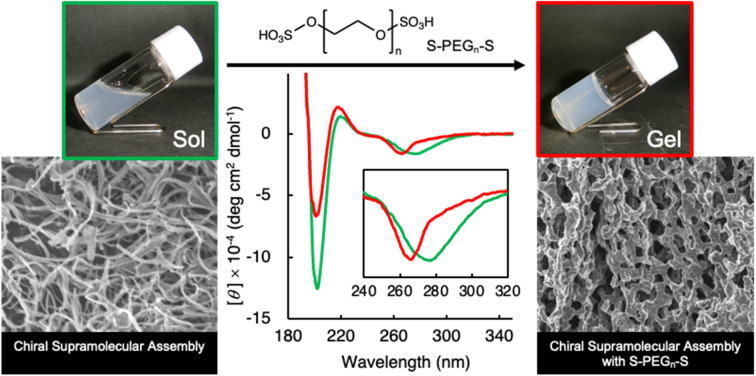

## Introduction

Self-healing artificial materials, which spontaneously self-heal any damage under ambient conditions, have attracted attention from researchers in recent times. In particular, polymer-based soft materials have been used to develop self-healing systems. These polymer network gels can exhibit drastic changes in their physical and thermal properties. Their self-healing involves the reconstruction of the chemical bonding to form a network structure consisting of covalent and non-covalent bond systems. Some examples of covalent bond systems are B–O bonds (*e.g.*, phenylboronate ester),^1^ S–S bonds (*e.g.*, disulfides),^2^ C–N bonds (*e.g.*, imines, acylhydrazones),^3,4^ C–C/C–S bonds (*e.g.*, reversible radical reactions),^5,6^ and cyclohexenes (*e.g.*, reversible Diels–Alder cycloaddition).^7,8^ Some examples of non-covalent bond systems are electrostatic interactions,^9^ ionic interactions,^10,11^ and hydrogen bonds.^12,13^ The chemical bonds link the polymer chains and exhibit dynamic and reversible properties. However, only a few examples of self-healing polymer network gels cross-linked with self-assembling surfactant-based micelles^14^ and bilayer membrane-based liposomes^15^ have been reported thus far. We previously reported self-assembled supramolecular hydrogels with cross-linked networks (hetero-network hydrogels), in which the polymer chains are linked with nanofibrous aggregates.^16,17^ In all such self-assembled cross-linked polymer hydrogels, the cross-linking points are formed by the insertion of hydrophobic groups such as cholesterol and long alkyl chains into the hydrophobic region of the self-assembly. The healing properties of such hydrogels originate from the co-aggregation of hydrophobic groups and self-assembling molecules. In this study, we used ionic interactions to cross-link a hydrophilic polymer with a supramolecular self-assembly. As the unique functions of supramolecular aggregates strongly depend on their molecular orientations, the bridging should not disturb the orientation structure of the self-assembly.

**Scheme 1:**
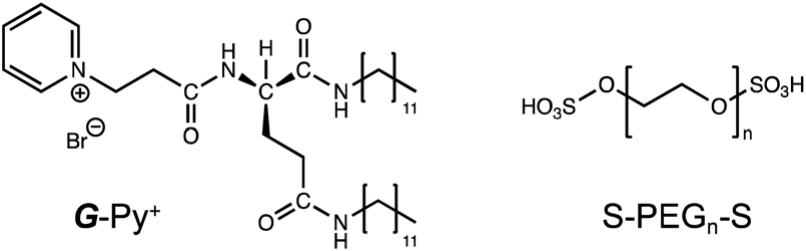
Chemical structures of glutamide-derived lipid with pyridinium head group (*G*-Py^+^) and termini-anionised hydrophilic polymer (S-PEG_*n*_-S).

Generally, ionic interactions have a smaller influence on the orientation structure than hydrophobic insertion because the bridging in the former case occurs at the surface of the self-assembly. In this study, we fabricated a network structure composed of a chiral supramolecular assembly of glutamide-derived lipids^18–20^ with pyridinium head groups (*G*-Py^+^) hybridised with termini-anionised hydrophilic polymers (S-PEG_*n*_-S). PEG was used as a nonionic water-soluble polymer that does not strongly interact with *G*-Py^+^. The formation of network hydrogels with one-dimensional (1D) nanostructures and a chiral self-assembly is expected to demonstrate self-healing functions. The chemical structures of *G*-Py^+^ and S-PEG_*n*_-S (*n* = average polymerisation degree provided by the supplier) are shown in Scheme 1.

## Experimental

### Materials

N-Benzyloxycarbonyl-l-glutamic (G-Z) acid and diethyl phosphorocyanidate (DEPC) were purchased from Tokyo Chemical Industry Co., Ltd (Tokyo, Japan). *N*-Dodecylamine and fluorescein were purchased from Kishida Chemical Co., Ltd (Osaka, Japan). Triethylamine, tetrahydrofuran, chloroform, hydrochloric acid, sodium sulfate, methanol, sodium hydroxide, diethyl ether, dichloromethane, acetone and isopentane were purchased from Nacalai Tesque Inc. (Kyoto, Japan). Ethanol, sodium hydrogen carbonate, Pd carbon, pyridine, chlorosulfonic acid were purchased from Fujifilm Wako Pure Chemical Co. (Osaka, Japan). 3-Bromopropionic acid was purchased from Sigma-Aldrich (Darmstadt, Germany).

Polyethylene glycol (PEG_*n*_) with different molecular weights (PEG_70_: 2700–3300, PEG_200_: 7300–9300, and PEG_350_: 15 000–25 000) were purchased from Fujifilm Wako Pure Chemical Co. (Osaka, Japan), Tokyo Chemical Industry Co., Ltd (Tokyo, Japan), and Nacalai Tesque Inc. (Kyoto, Japan), respectively. All chemicals were used without further purification.

### Synthesis of *G*-Py^+^ and S-PEG_*n*_-S

*G*-Py^+^ was synthesised using a previously described procedure,^21^ and its chemical structure was confirmed *via* melting point determination (MP-500P, Anatec Yanaco Co. (Kyoto, Japan)), elemental analysis (Micro Corder JM10, J Science Lab. Co. Ltd (Kyoto, Japan)), Fourier-transform infrared (FT-IR) spectroscopy (Fig. S1;† FT/IR-4100, JASCO Co. (Tokyo, Japan)), and ^1^H NMR spectroscopy (Fig. S2 and S3;† JNM-ECZ400R, JEOL Ltd (Tokyo, Japan)).

S-PEG_*n*_-S was prepared by sulfation of the terminal hydroxyl groups of PEG_*n*_ using chlorosulfonic acid.^22^ The details are described in the ESI.†

### Synthesis of hydrogels containing *G*-Py^+^ and S-PEG_*n*_-S

*G*-Py^+^ was dissolved in water *via* ultrasonication (Model Q500, Qsonica, CT, USA) for 5 min, aged at 25 °C for 30 min, and allowed to stand at 10 °C for 1 h. S-PEG_*n*_-S was added to the solution to obtain varying concentrations, and the mixture was vortexed for 1 min. The mixture was heated in a water bath at 60 °C for 30 min and allowed to stand at 10 °C for 1 h.

### Characterisation

#### Gelation

The gelation properties of the hydrogels of various concentrations were evaluated in a sample tube with a 10 mm inner diameter *via* the fluid inversion method.

#### Scanning electron microscopy (SEM)

The hydrogels were incubated in acetone with dry ice. The sample tubes of hydrogels were immersed in an aluminium container containing isopentane, dry ice, and acetone and pre-frozen for 1 min. The temperature of the isopentane solution was −75 °C. The samples were freeze-dried for 24 h using a freeze-dryer. The freeze-dried samples were coated with Os using an osmium coater (OPC60A, Filgen Inc., Nagoya, Japan) and then observed through the SEM microscope (JCM-5700, JEOL Ltd, Tokyo, Japan).

#### Transmission electron microscopy (TEM)

Hydrogels were prepared in sample tubes using 10 mM *G*-Py^+^ and 6 wt% S-PEG_200_-S using the above-mentioned synthesis method. The gels were cast onto a carbon-deposited, ion-coated copper mesh using a capillary tube. Subsequently, after 30 s, the water was absorbed by the filter paper, which was then dried under reduced pressure for 3 h. The casted samples were stained with 1 wt% of uranyl acetate (UO_2_^2+^) aqueous solution and observed through the TEM microscope (JEM-1400Plus, JEOL Ltd, Tokyo, Japan).

#### Confocal laser scanning microscopy (CLSM)

A mixed aqueous solution of 0.5 mM *G*-Py^+^ and 1 wt% S-PEG_200_-S was prepared using the above-mentioned synthesis method. Sulforhodamine B, an anionic fluorescent dye, was added to this solution and dropped onto a cover glass, which was placed on the sample table of a confocal laser microscope. A CLSM microscope (TSC SP3, Leica Microsystems GmbH, Wetzlar, Germany), with a 552 nm laser for excitation, was used to observe the *G*-Py^+^ assembly.

#### Circular dichroism (CD)

The CD spectra of the *G*-Py^+^ solution in a 1 mm quartz cell were measured using the CD spectrophotometer (J-725, JASCO Co. Ltd, Tokyo, Japan). Conditions: measurement sensitivity: standard; measurement temperature: 10 °C; start wavelength: 400 nm; end wavelength: 200 nm; scanning mode: continuous; scanning speed: 200 nm min; response: 2.0 s; bandwidth: 1.0 nm; and integration frequency: 1 time.

#### Thermal analysis

An aqueous *G*-Py^+^ solution (10 mM) was ultrasonicated for 5 min, and different amounts of S-PEG_*n*_-S were added to the solution. Thermograms of the *G*-Py^+^ assembly in an aqueous solution in the presence and absence of S-PEG_*n*_-S were recorded in a sealed silver cell at a heating rate of 2 °C min^−1^ using a differential scanning calorimeter (DSC) (DSC7000X, Hitachi High-Tech Co., Tokyo, Japan).

#### Rheological properties

The prepared hydrogels were heated in a water bath at 60 °C to a sol state, and three drops were placed on the Peltier plate of the rheometer (Discovery, TA Instruments, New Castle, USA). The measurements were taken with a cone geometry of 1° at a wide range of temperatures from 10 to 50 °C.

## Results and discussion

### Formation of the self-healable hydrogels

**Fig. 1:**
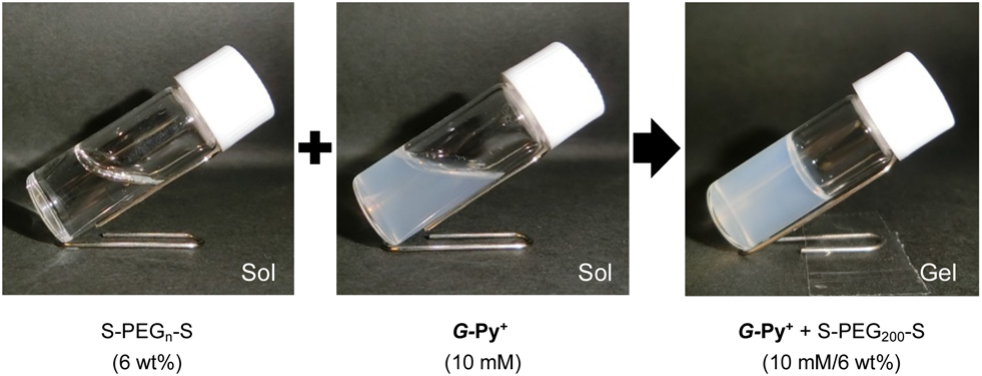
Aqueous solutions of *G*-Py^+^ (10 mM), S-PEG_*n*_-S (6 wt%), and *G*-Py^+^ (10 mM)/S-PEG_*n*_-S (6 wt%).

The aqueous solutions of *G*-Py^+^ and S-PEG_*n*_-S (*n* is the average polymerisation degree) were mixed, heated to 60 °C, aged for 30 min, cooled to 25 °C, and aged for 1 h. Photographs of the aqueous solutions of *G*-Py^+^ (10 mM), S-PEG_200_-S (6 wt%), and *G*-Py^+^/S-PEG_200_-S (10 mM/6 wt%) are shown in Fig. 1. The aqueous solution of *G*-Py^+^ was pale white due to the nanofibrous self-assemblies with 20–30 nm diameter and a large aspect ratio (described later in the paper). No gelation was observed in the *G*-Py^+^ and S-PEG_200_-S solutions, whereas gelation was observed in the aqueous mixture of *G*-Py^+^ and S-PEG_200_-S. The *G*-Py^+^/S-PEG_200_-S hydrogel was turbid, suggesting the preserving of nanofibrous aggregates even after the addition of S-PEG_200_-S. These results indicate that the gelation was induced through the interactions between fibrous aggregates of *G*-Py^+^ and S-PEG_*n*_-S components. As no gelation was observed in the *G*-Py^+^ assembly with PEG (without anionic termini), the ionic interactions between the pyridinium group of *G*-Py^+^ and the sulfate group of S-PEG_200_-S were deemed essential for hydrogel formation. This gelation behaviour was confirmed through the inversion fluid method for the aqueous mixtures of *G*-Py^+^ and S-PEG_*n*_-S with different polymerisation degrees.

As shown in Table 1, the gelation behaviour was affected by the concentrations of *G*-Py^+^ and S-PEG_*n*_-S. Higher concentrations of *G*-Py^+^ and S-PEG_*n*_-S tended to form gel, which was common in polymer network gels. Interestingly, the gelation ability also depended on the polymerisation degree of S-PEG_*n*_-S. These results suggest that an appropriate balance between the polymer chain length and the terminal interaction sites is required for gelation. This inference was confirmed by the absence of gelation in an aqueous mixture of 10 mM *G*-Py^+^ and 6 wt% S-PEG_70_-S, whereas gelation was observed with 1 wt% S-PEG_70_-S. Moreover, the orientation structure of *G*-Py^+^ was affected by the number of sulfate groups (described later in the paper).

**Table 1:** Gelation properties of various mixtures of *G*-Py^+^ and S-PEG_*n*_-S^a^

*G*-Py^+^	S-PEG_70_-S		S-PEG_200_-S		S-PEG_350_-S	
	1 wt%	6 wt%	1 wt%	6 wt%	1 wt%	6 wt%
1 mM	S	S	S	S	S	S
5 mM	S	S	G	G	S	G
10 mM	G	S	G	G	S	G

a S: solution state; G: gel state.

### Rheological properties of hybrid hydrogels

The strain dependence of the elastic storage moduli (*G*′) and the viscous loss moduli (*G*′′) for an aqueous mixture of *G*-Py^+^ and S-PEG_*n*_-S (10 mM/6 wt%) at 10 °C with a wide range of oscillation strain from 0.01% to 100% is shown in Fig. S4.† Both *G*′ and *G*′′ were stable at less than 0.25% oscillation, and *G*′ was always higher than *G*′′ in this range, indicating hydrogel formation.

**Fig. 2:**
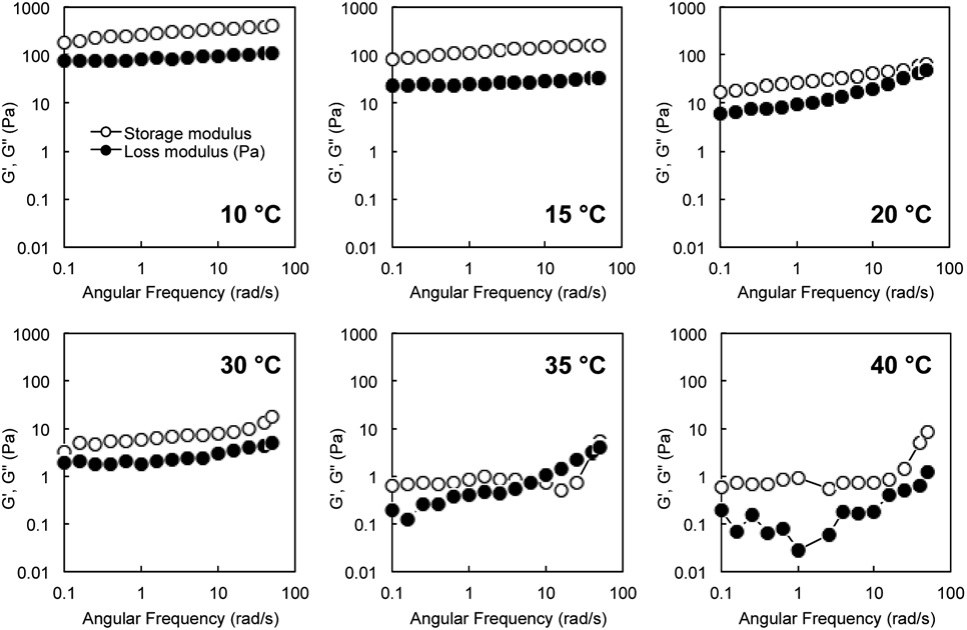
Angular frequency dependence (rad s^−1^) of storage modulus *G*′ (open circles) and loss modulus *G*′′ (closed circles) of *G*-Py^+^/S-PEG_200_-S (10 mM/6 wt%) hybrid hydrogels at various temperatures and 0.25% strain.

Rheological measurements of *G*-Py^+^/S-PEG_200_-S hydrogels revealed a single plateau region in the dynamic moduli as functions of angular frequency (0.1–60 rad s^−1^) at a fixed strain (*γ* = 0.25%). *G*′ had a substantial elastic response and was always larger than *G*′′. Fig. 2 shows the angular frequency dependencies of *G*′ and *G*′′ of the hybrid hydrogels at various temperatures. The single plateau observed from these viscoelastic measurements at lower temperatures revealed a well-developed network. However, high *G*′ and *G*′′ were not observed for the *G*-Py^+^ solution, which suggests that S-PEG_200_-S cross-linked with the fibrous self-assembly of *G*-Py^+^ in the *G*-Py^+^/S-PEG_200_-S solution to form network structure. Furthermore, *G*′ correlates with the rigidity of hydrogels, which suggests that the hybrid hydrogels are strong gels at lower temperatures. *G*′ and *G*′′ values of the aqueous mixture of *G*-Py^+^/S-PEG_200_-S (10 mM/6 wt%) indicate that the mixture was in a gel state below 30 °C. Both values decreased slightly with increasing temperature, suggesting that the gel softens at higher temperatures. At 35 °C, *G*′ and *G*′′ values drastically decreased to <10 Pa and were reversed in the low-frequency region. The values of *G*′ and *G*′′ decreased as temperature increased, and became very small above 35 °C which is almost identical to the gel-to-liquid crystal phase transition temperature (*T*_C_) of *G*-Py^+^. These results strongly support that a mixed aqueous solution of *G*-Py^+^ and S-PEG_200_-S forms a 3D network gel when *G*-Py^+^ is in the gel state (temperature < phase transition temperature), but is in a solution state when *G*-Py^+^ is in the liquid crystal state (temperature > *T*_C_). As these changes are reversible, we conclude that the gel-to-solution changes in the aqueous solution are induced by the gel-to-liquid crystalline phase transition of *G*-Py^+^.

### Network structure of the nanotubular self-assembly

**Fig. 3:**
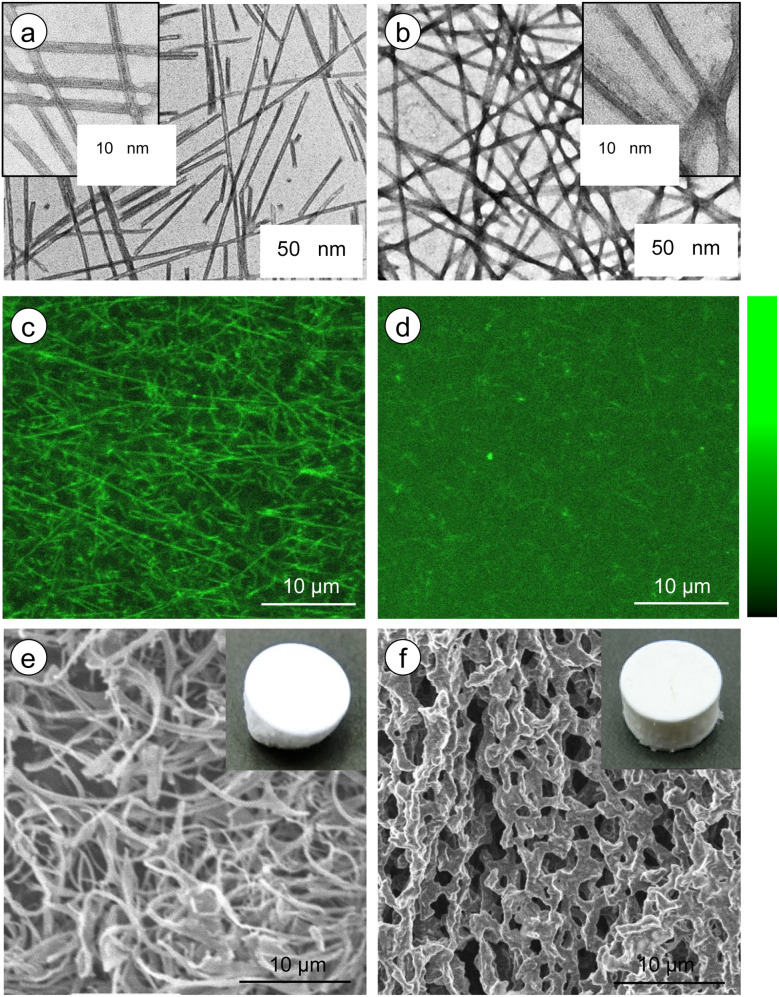
(a and b) Transmission electron microscopy and (e and f) scanning electron microscopy images of *G*-Py^+^ (10 mM) self-assemblies without and with S-PEG_200_-S (6 wt%). For TEM observations, 1 wt% of UO_2_^2+^ was used for staining. For SEM observations, the aqueous solution was freeze-dried, and the samples were coated with osmium. (c and d) Confocal laser scanning microscopy images of *G*-Py^+^ (0.5 mM) self-assemblies without and with S-PEG_200_-S (1 wt%). Sulforhodamine B was used as the fluorescent reagent, and the excitation wavelength was 552 nm. Brightness (color bar) are scaled 0 to 255.

Microscopic observations were conducted to confirm the aggregation morphology of the *G*-Py^+^ assemblies with and without S-PEG_*n*_-S. In an aqueous solution, *G*-Py^+^ formed nanotubular aggregates based on a bilayer membrane structure. The inner spaces of the nanotubes were clearly observed on the TEM image of the only *G*-Py^+^ (Fig. 3a) by negative staining with UO_2_^2+^. The inner and outer diameters of the tubular aggregates were 7.3 and 12.6 nm, respectively, and the thickness of the bilayer membrane structure was 2.6 nm, which corresponds to the length of two *G*-Py^+^ molecules. In contrast, the inner spaces of the nanotubular aggregates was difficult to detect on the *G*-Py^+^/S-PEG_200_-S (Fig. 3b). It is considered that S-PEG_200_-S coated nanotube aggregates of *G*-Py^+^, but perturbation to the aggregate morphology was small because the tubular morphology was maintained. Furthermore, a slightly dark silhouette was observed around the fibrous aggregates of *G*-Py^+^/S-PEG_200_-S, which was not observed for the only *G*-Py^+^. These silhouettes are attributed to the polymer chains of S-PEG_200_-S. As the polymer backbone is hydrophilic and non-ionic, the polymer chains might have been slightly stained by UO_2_^2+^.

Similar results were obtained from the CLSM images of *G*-Py^+^ (Fig. 3c and d, the brightness profiles are shown in Fig. S5†) stained with sulforhodamine B, an anionic fluorescent dye. A fibrous shape was observed in the emission image of *G*-Py^+^. The anionic dye is expected to be concentrated on the surface of the nanotubular aggregates through ionic interactions, which is revealed as emission around the nanotubes under laser irradiation (552 nm). No such emission of fibrous aggregates was observed for *G*-Py^+^/S-PEG_200_-S, indicating that the PEG chains may have covered the nanotubes, thus preventing the concentration of dye on them. The SEM observations of freeze-dried *G*-Py^+^ without (Fig. 3e) and with S-PEG_200_-S (Fig. 3f) confirmed that the polymer coated *G*-Py^+^ assembly. Similar observations were not obtained for the *G*-Py^+^ assembly with PEG_200_ having no anionic terminal groups, indicating that PEG_200_ does not cover thickly with *G*-Py^+^ assemblies. These observations also indicate that the terminal sulfate groups of S-PEG_200_-S trigger the polymer coating of the *G*-Py^+^ assembly. When PEG_200_ was added to *G*-Py^+^, a fibrous structures were observed (Fig. S6†). This result indicates that PEG_200_ may not concentrate on the *G*-Py^+^ nanofibrous aggregates and dissolves uniformly in the solution.

### Ordered structure of the *G*-Py^+^ self-assembly

**Fig. 4:**
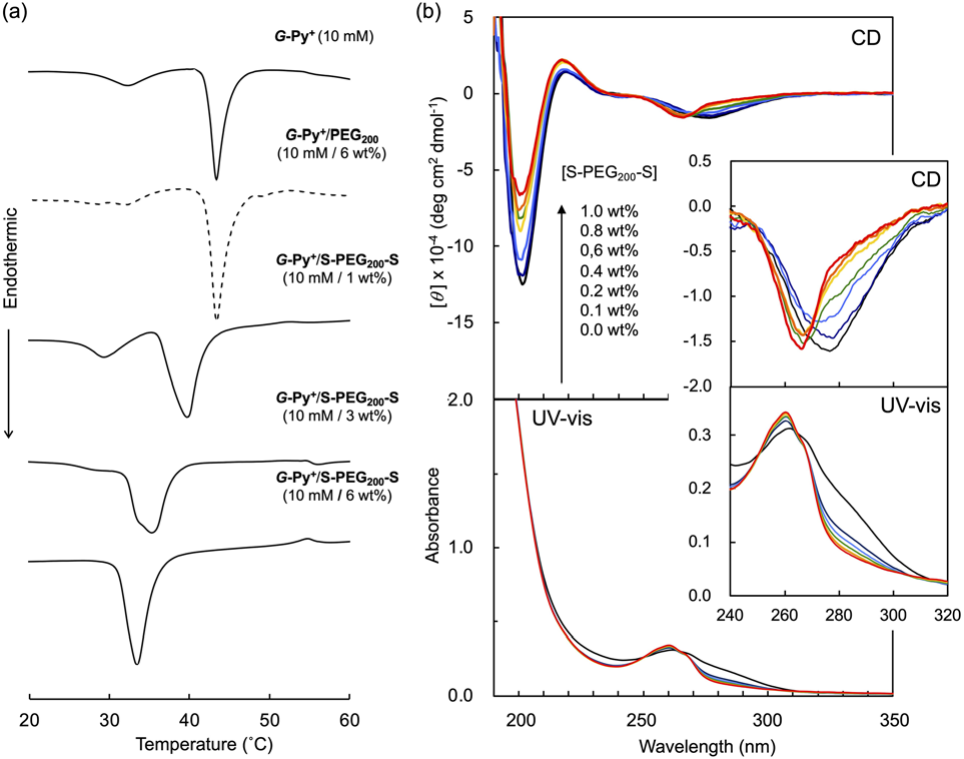
(a) Differential scanning calorimetry (DSC) thermograms and (b) circular dichroism (CD) and absorption (UV-vis) spectra of the aqueous solutions of *G*-Py^+^ without and with S-PEG_200_-S. DSC analysis: [*G*-Py^+^] = 10 mM; [S-PEG200-S] = 0, 1, 3, and 6 wt%. CD and UV-vis measurements: [*G*-Py^+^] = 0.5 mM; [S-PEG_200_-S] = 0, 0.1, 0.2, 0.4, 0.6, 0.8, and 1.0 wt%.

**Fig. 5:**
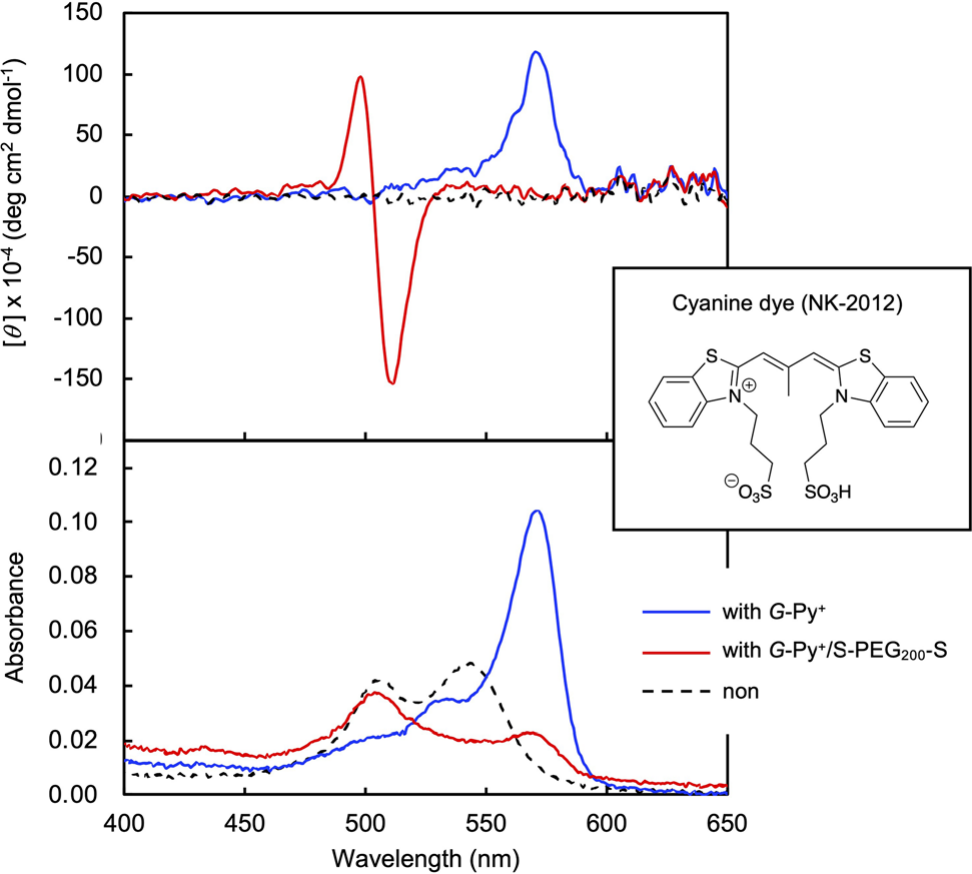
Circular dichroism (CD) and absorption (UV-vis) spectra of cyanine dye (NK-2012) in the aqueous solutions in the absence and the presence of *G*-Py^+^ and *G*-Py^+^/S-PEG_200_-S. [NK-2012] = 0.0125 mM; [*G*-Py^+^] = 0.5 mM; [S-PEG_200_-S] = 1.0 wt%.

In the DSC thermogram of the aqueous solution of *G*-Py^+^, endothermic peaks with peak top temperatures of 33 °C and 43 °C were observed (Fig. 4a). According to our previous study, these peaks are related to the gel-to-gel phase transition involving inversion of the chiral orientation of amide bonds and gel-to-liquid crystalline phase transition of the alkyl chains of *G*-Py^+^, respectively. These phase transition temperatures decreased with increasing concentrations of S-PEG_200_-S. However, the enthalpy (Δ*H*) of the phase transitions remained constant, even with a large amount of S-PEG_200_-S. The Δ*H* values of the main transitions without and with 6 wt% S-PEG_200_-S were 22.4 and 32.7 kJ mol^−1^, respectively. Notably, the highly-oriented structure of *G*-Py^+^ was maintained despite the accompanying decrease in the phase transition temperature. Since no change was observed in the phase transition behaviour (*T*_C_ and Δ*H*) of *G*-Py^+^ aggregates with PEG_200_, we conclude that the polyethylene chains do not affect the ordered structure of *G*-Py^+^. These results also support the assumption that the anionic terminal sulfate groups of the polymer interacted with the cationic pyridinium group of the *G*-Py^+^ self-assembly. We have reported that *in situ* polymerisation of styrene in the hydrophobic field of *G*-Py^+^ bilayer membranes results in a decrease in the *T*_C_ without any change in Δ*H*.^23^ It was assumed that the polystyrene formed in *G*-Py^+^ self-assembly intercalated into the lipid layers, changing its ordered structure into a different stable form without disruption. We also reported that the addition of polyethylene glycol terminated with octadecyl alkyl groups to *G*-Py^+^ self-assembly causes a simultaneous decrease in temperature and Δ*H* of the phase transition of *G*-Py^+^.^16^ In this case, the terminal alkyl chains inserted into lipid bilayer, inducing the disordering of molecular orientation of *G*-Py^+^. CD spectroscopic measurements supported this prediction (Fig. 4b). The *G*-Py^+^ aqueous solution (0.5 mM) exhibited large CD signals at 202 nm ([*θ*] = −12.3 × 10^4^ deg cm^2^ dmol^−1^) and 280 nm ([*θ*] = −1.6 × 10^4^ deg cm^2^ dmol^−1^). These CD signals correspond to the amide bond and the pyridinium group (UV-vis spectra are shown in Fig. 4b), respectively, and enhance with chiral stacking. Upon the addition of S-PEG_200_-S, the CD signal of the pyridinium group shifted from 280 to 268 nm without a intensity without wavelength change with the addition of decrease in signal intensity. Moreover, the intensity ratio of the positive and negative CD signals around 200 nm, which is related to amide bonds, changed. However, the CD signal intensities were mostly retained, even with excess S-PEG_200_-S. Previous studies reported that the CD signals of chromophore groups in the gel state of bilayer membranes remarkably decrease in the liquid-crystalline state (at a temperature above *T*_C_). The CD signal with a peak top at 201.5 nm ([*θ*] = −12.5 × 10^4^ deg cm^2^ dmol^−1^), which was based on the torsion of intermolecular hydrogen bond of the amide groups, gradually decreased inpolymer. The CD signal ([*θ*] = −1.61 × 10^4^ deg cm^2^ dmol^−1^) with a peak top at 276.5 nm, which was based on the chiral stacking of the pyridinium group, blue-shifted to 266 nm with the addition of the polymer. These results suggested that the surface polarity of the chiral supramolecular assembly of *G*-Py^+^ was likely altered by the addition of S-PEG_200_-S, but the effect on the chiral oriented structure is not significant. To discuss the change in the orientation structure of *G*-Py^+^ by the addition of S-PEG_200_-S in more detail, an anionic cyanine dye (NK-2012) was used as an indicator. The absorption peaks of NK-2012 appeared at 504 nm and 540 nm which correspond to the dye in dimeric *H*-aggregate and monomeric state respectively, and blue-shifted to 570 nm when added to the aqueous solution of *G*-Py^+^ assemblies. A significantly large CD signal ([*θ*]_571_ = +11.8 × 10^5^ deg cm^2^ dmol^−1^) was observed around the absorption peak, suggesting that NK-2012 formed chiral *J*-aggregates through the complexation with *G*-Py^+^ assemblies.^19,20,24^ On the other hand, when NK-2012 was added to the *G*-Py^+^/S-PEG_200_-S aqueous mixture, the absorption around 506 nm increased and the significantly large CD signal with Cotton effect ([*θ*]_498_ = +9.66 × 10^5^ deg cm^2^ dmol^−1^, [*θ*]_512_ = −15.1 × 10^6^ deg cm^2^ dmol^−1^) was exhibited at the corresponding wavelength. It is surprising that the addition of S-PEG_200_-S to the *G*-Py^+^ assembly significantly affected the chiral orientation structure of the complex-forming dye, even though the change in the CD spectrum of *G*-Py^+^ is small (shown in Fig. 5). Further investigation is needed to elucidate the details of these phenomena.

### Self-healing property based on the cross-linking of the polymer network with self-assembled nanotubular aggregates

**Fig. 6:**
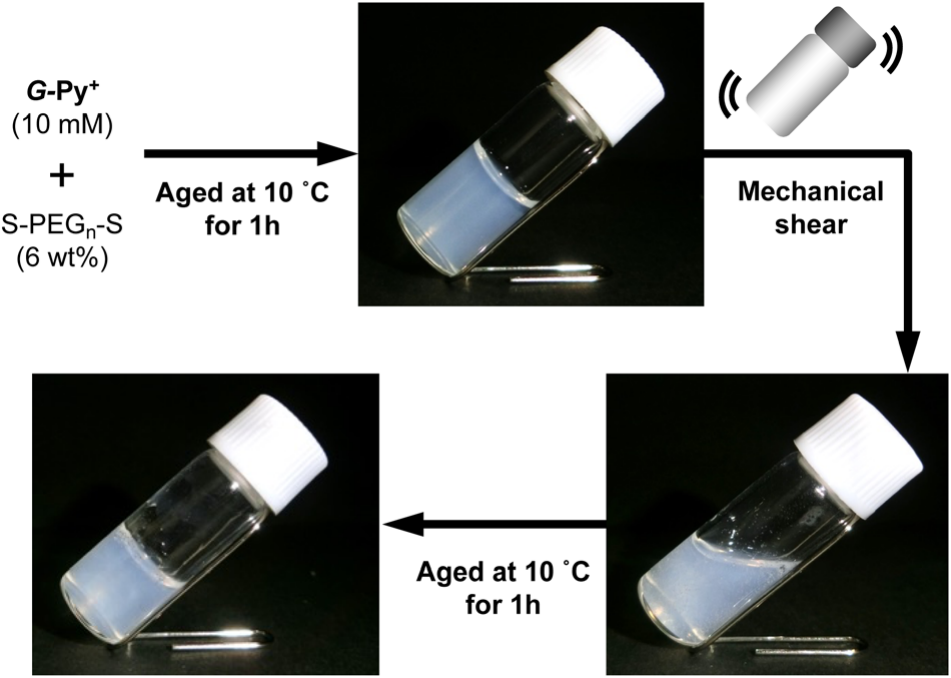
Self-healing property of the hydrogel formed from an aqueous mixture of *G*-Py^+^ and S-PEG_200_-S at 10 °C. The hydrogel was mechanically disruption by vortexing. [*G*-Py^+^] = 10 mM; [S-PEG_200_-S] = 6 wt%.

The hybrid hydrogel is formed through the cross-linking of the polymer network with the self-assembled nanotubular aggregates. This would lead to a spontaneous rearrangement of the network structure, which is considered self-healing or self-recovering. The *G*-Py^+^/S-PEG_200_-S (10 mM/6 wt%) hetero-network hydrogel was mechanically broken by vortexing and was allowed to stand at varying temperatures for 1 h. As shown in Fig. 6, the gel state was recovered after ageing at 10 °C for 1 h. Similar recovering behaviour was observed in broken hydrogels after ageing at 15 °C and 25 °C for 1 h, and this self-healing behaviour was reproducible, but was not observed at 35 °C (Fig. S7†). These results suggest that the self-healing of the hydrogel is related to the phase transition of the *G*-Py^+^ aggregates. At ≤25 °C, which is sufficiently below the phase transition temperature, the gel is regenerated. However, at temperature above *T*_C_ of the *G*-Py^+^ aggregates, the lipid molecules remained in a liquid crystalline state suggesting that the gel could not be regenerated due to the loose packing of the lipid molecules. The mechanical disruption can lead to fragmentation of nanofibrous aggregates, which induces the aqueous mixture into a sol state. It is anticipated that the self-healing is induced by the recovery of supramolecular nanofibers at temperatures below *T*_C_, but is not occurred at temperature above *T*_C_ due to the difficulty in recovery of the nanofibrous aggregates.

**Fig. 7:**
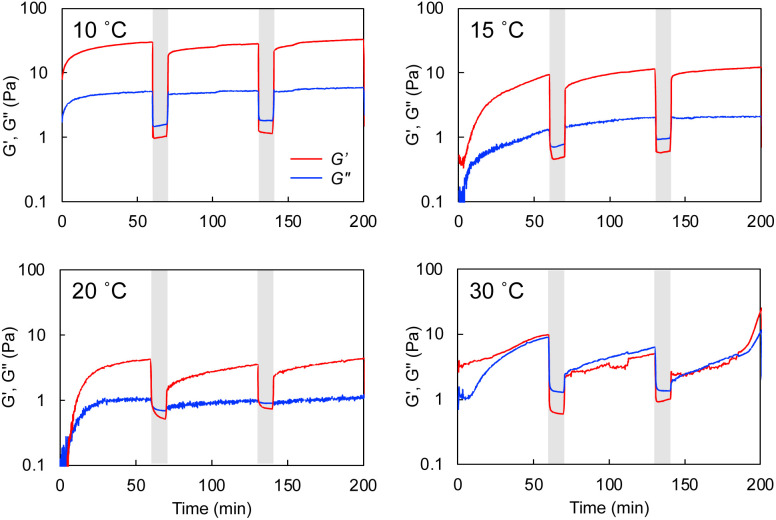
Rheological results of the self-healing property of the *G*-Py^+^/S-PEG_200_-S hydrogel at various temperatures. The hydrogel was broken by oscillation with a large strain of 25% (grey marker). Measurement oscillation strain: 0.25%. [*G*-Py^+^] = 10 mM; [S-PEG_200_-S] = 6 wt%.

Rheological experiments also proved the gel-recovering (self-healing) property of the aqueous hybrid hydrogels. A warm aqueous mixture (sufficiently higher temperature than *T*_C_) of *G*-Py^+^ and S-PEG_200_-S was placed on the rheometer stage, cooled to a given temperature, and aged for 1 h before the measurement. As shown in Fig. 7, *G*′ was larger than *G*′′ at a fixed strain (*γ* = 0.25%) at 10 °C. When the strain was increased to 25%, both *G*′ and *G*′′ decreased, and *G*′ was lower than *G*′′, indicating that the mixture changed from a gel to a solution state. The *G*′ and *G*′′ (gel state) were quickly recovered when the strain was decreased to 0.25%. Similar phenomena were observed at 15 and 20 °C with lower *G*′ and *G*′′. However, the *G*′ and *G*′′ values were the smallest thus far and comparable at 30 °C. Hence, the hydrogels self-healed after removing the strain at *T* < *T*_C_. As the temperature increased, *G*′ and *G*′′ values became smaller, which indicates softening of the gel. Similar gel recovery was repeatedly observed at 15 and 20 °C. However, *G*′ and *G*′′ values were almost identical at 30 °C, and no differences were observed after a mechanical breakdown *via* vortexing. These results support the gel-recovery behaviour observed through the inversion method, and we conclude that the *G*-Py^+^ phase transition affects the self-healing property of the hydrogel. The phase transition of the glutamide lipids varies with the alkyl chain length and the chemical structure of the functional head group, which suggests that the mechanical strength and the recovering temperature of a hybrid hydrogel containing a fibrous self-assembly and a polymer can be fine-tuned by changing the chemical structures of the lipids and the polymer chain length (*M*_w_). The self-healing temperature could be controlled by changing these parameters in the chemical structures.

## Conclusions

Hybrid hydrogels based on polymer-tangled nanotubular self-assembly were successfully fabricated. The terminal anionic groups of S-PEG_200_-S interacted with the cationic pyridinium groups of *G*-Py^+^ and triggered the tangling of the PEG main chains with the nanotubular self-assembly. The chiral orientation of *G*-Py^+^ among the bilayer membranes was arranged in a different chiral orientation in the presence of excess S-PEG_200_-S, but the morphology of the tubular aggregates was preserved. The hybrid hydrogels exhibited self-healing at a temperature below *T*_C_, which is based on the gel state of the bilayer membranes in the supramolecular chiral self-assemblies.

## Data availability

The data supporting this article have been included as part of the ESI.†

## Author contributions

Makoto Takafuji: conceptualization, methodology, project administration, resources, supervision, validation, writing – original draft, visualization; Kenji Kawamoto: investigation, formal analysis, validation; Nanami Hano: data curation, investigation, visualization, writing – review & editing; Mako Otsuki: investigation, visualization; Hirotaka Ihara: resources, supervision, writing – review & editing.

## Conflicts of interest

There are no conflicts to declare.

## Supplementary Material

NA-006-D4NA00353E-s001
