# Supplementary material for: Nanofibrous chiral supramolecular assembly-derived self-healing hydrogels with polyethylene glycol
Source: Nanoscale Adv. 2024 Jun 10;6(15):3850–6. doi: 10.1039/d4na00353e (PMC11265573; doi:10.1039/d4na00353e)
Supplement: NA-006-D4NA00353E-s001 [file NA-006-D4NA00353E-s001.pdf]

Electronic Supplementary Material (ESI) for Nanoscale Advances.
This journal is © The Royal Society of Chemistry 2024

Supplementary Information

Nanofibrous Chiral Supramolecular Assembly-Derived Self-Healing Hydrogels With Polyethylene Glycol

Makoto Takafuji,*[a,b] Kenji Kawamoto,[a] Nanami Hano,[a,b]
Mako Otsuki,[a] and Hirotaka Ihara*[a,b]

[a] *Faculty of Advanced Science and Technology, Kumamoto University, 2-39-1 Kurokami, Chuo-ku, Kumamoto 860-8555, Japan.*

[b] *International Research Organization for Advanced Science and Technology (IROAST), Kumamoto University, 2-39-1 Kurokami, Chuo-ku, Kumamoto 860-8555, Japan.*

Tel: +81-96-342-3661; E-mail: takafuji@kumamoto-u.ac.jp, ihara@kumamoto-u.ac.jp

Synthesis of *G*-Py^{+}

Synthesis scheme of *G*-Py^{+} was depicted and the synthesis of each step is described below.

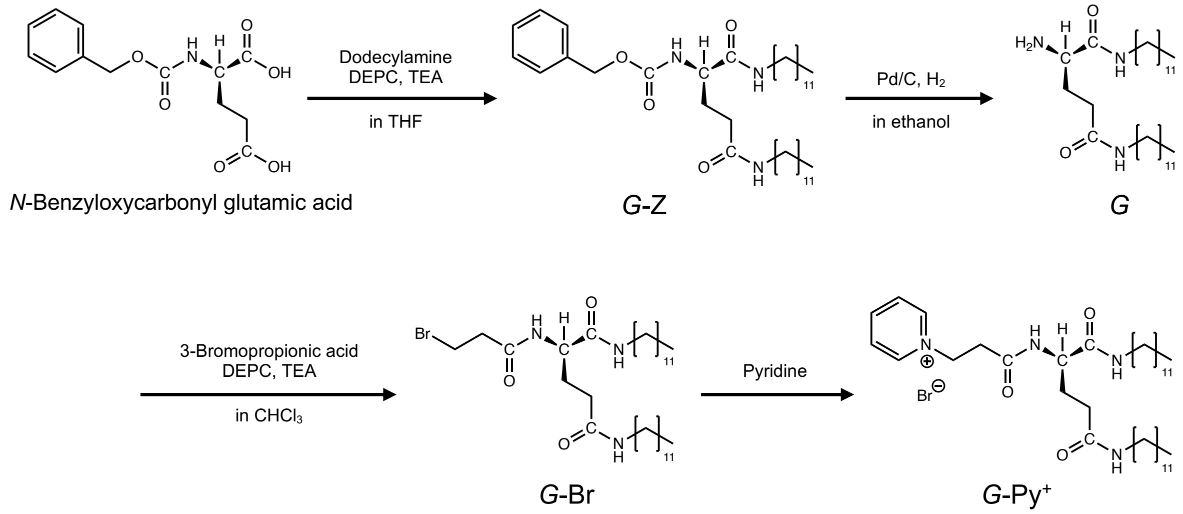

N',N''-Didodecyl-*N*-benzyloxycarbonyl glutamide (*G*-Z).

N-Benzyloxycarbonyl glutamic acid (6.02 g, 21.4 mmol), *n*-dodecylamine (9.14 g, 49.3 mmol), and triethylamine (5.34 g, 52.8 mmol) were dissolved in THF (300 mL). The solution was cooled to 0 °C, and diethyl phosphorocyanidate (DEPC) (8.01 g, 49.1 mmol) was added to the solution and stirred for 1 h at this temperature. After stirring for 1 day at room temperature, the solution was concentrated *in vacuo*, and the residue was dissolved in 300 mL of chloroform. The solution was washed with 5wt% $NaHCO_3$, 0.3 mol/L HCl, and water. The solution was dried over Na_2SO_4, concentrated, and finally recrystallized from ethanol, which gave a white solid powder: yield 9.48 g (72.6 %, 15.4 mmol); mp 140.8-141.8 °C; FT-IR (KBr) / 3289, 2917, 2850, 1686, 1646 and 1558 cm^{-1}; ^{1}H-NMR ($CDCl_3$)δ 0.88 (t, 6H, CH_3), 1.26 (br s, 36H, $(CH_2)_9CH_3$), 1.46-1.48 (m, 4H, $CONHCH_2CH_2$), 1.93-2.11 (m, 2H, C^*HCH_2), 2.24-2.42 (m, 2H, $C^*HCH_2CH_2$), 3.20-3.26 (m, 4H, $CONHCH_2$), 4.10-4.20 (t, 1H, C*H), 5.06-5.15 (b, 2H, CH_2Ph), 7.29-7.45 (m, 5H, aromatics).

N',N''-Didodecyl glutamide (*G*).

G-Z (7.05 g, 11.4 mmol) was dissolved in 300 mL ethanol with heating, and Pd carbon (0.54 g) was added to the solution. H_2 gas was bubbled slowly into the solution for 6 h at 60 °C. Pd carbon was removed by filtration. The solution was concentrated, recrystallized from methanol, and dried *in vacuo* to give a white solid powder: yield 3.40 g (61.7 %, 7.06 mmol); mp 115.9-117.0 °C; FT-IR (KBr) / 3326, 2920, 2850, 1634, and 1532 cm^{-1}; ^{1}H-NMR ($CDCl_3$) : δ 0.88 (t, 6H, CH_3), 1.26 (br s, 36H, $(CH_2)_9CH_3$),

1.46-1.54 (m, 4H, $CONHCH_2CH_2$), 1.93-1.97 (m, 2H, C^*HCH_2), 2.27-2.38 (m, 2H, $C^*HCH_2CH_2$), 3.20-3.26 (m, 4H, $CONHCH_2$), 3.42-3.46 (t, 1H, C*H).

N',N''-Didodecyl-*N*-bromopropionyl glutamide (*G*-Br).

G (2.29 g, 4.75 mmol) was dissolved in chloroform (100 mL); 3-bromopropionic acid (0.93 g, 6.08 mmol) DEPC (1.06 g, 6.54 mmol) and triethylamine (1.21 g, 11.9 mmol) were also added into the solution. The solution was cooled to 0 °C and stirred for 1h at this temperature. After being stirred for 1 day at room temperature, the solution was concentrated, and the residue was dissolved in chloroform. The solution was washed with 1.0 mol/L HCl, 1.0 mol/L NaOH and water. The solution was dried over Na_2SO_4, concentrated, and finally recrystallized from ethanol, which gave a white solid powder: yield 1.64 g (55.9 %, 2.66 mmol); mp 157.2-160.0 °C; FT-IR (KBr) / 3292, 2920, 2850, 1639, and 1556 cm^{-1}; ^{1}H-NMR ($CDCl_3$) : δ 0.88 (t, 6H, CH_3), 1.26 (s, 36H, $(CH_2)_9CH_3$), 1.43-1.56 (m, 4H, $CONHCH_2CH_2$), 2.00-2.13 (m, 2H, C^*HCH_2), 2.28-2.51 (m, 2H, $C^*HCH_2CH_2$), 2.80-2.83 (m, 2H, CH_2CH_2Br), 3.20-3.29 (m, 4H, $CONHCH_2$), 3.66 (t, 2H, CH_2Br), 4.36-4.43 (t, 1H, C*H).

N',N''-Didodecyl(pyridinium-*N*-propionyl)glutamide Bromide (*G*-Py^+)

G-Br (0.50 g, 0.81mmol) was dissolved in 50 mL of pyridine and stirred at reflux temperature for 2 days. After cooling to room temperature, a white crystal appeared, and unreacted pyridine was removed by filtration. The white crystals in this step were dried *in vacuo*: yield 0.22 g (39 %, 0.32 mmol); mp 180.9-183.9 °C; FT-IR (KBr) / 3302, 2917, 2850, 1646, and 1560 cm^{-1}; ^{1}H-NMR ($CDCl_3$) : δ 0.88 (t, 6H, CH_3), 1.25 (br s, 36H, CH_2), 1.45-1.50 (m, 4H, CH_2), 1.91-2.11 (m, 2H, C^*HCH_2), 2.21-2.35 (m, 2H, $C^*HCH_2CH_2$), 5.15-5.19 (t, 2H, (Py)CH_2CH_2CONH), 6.84 (t, 1H, NH), 6.94 (t, 1H, NH), 8.05-8.07 (t, 1H, m-Py), 8.35-8.36 (d, 1H, NH), 8.41-8.45 (t, 1H, p-Py), 9.40-9.44 (d, 1H, o-Py). FT-IR and ^{1}H NMR spectra were shown in Figure S1 and S2.

Figure S1

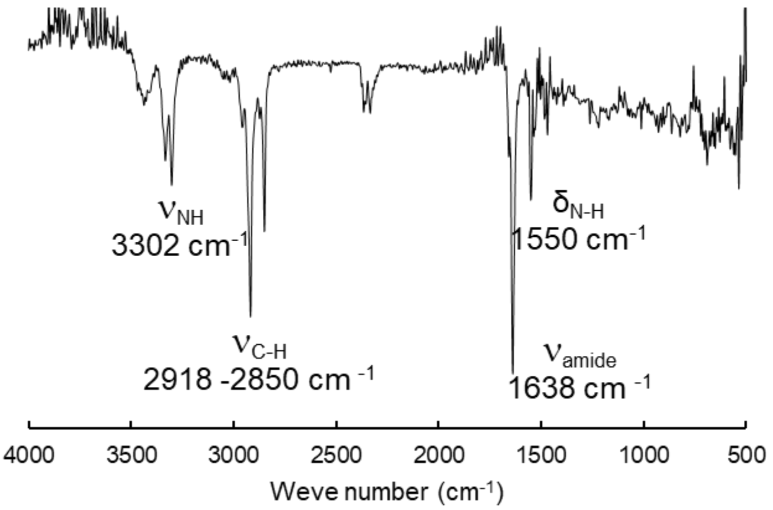

Figure S1 FT-IR spectrum of *G*-Py+.

Figure S2

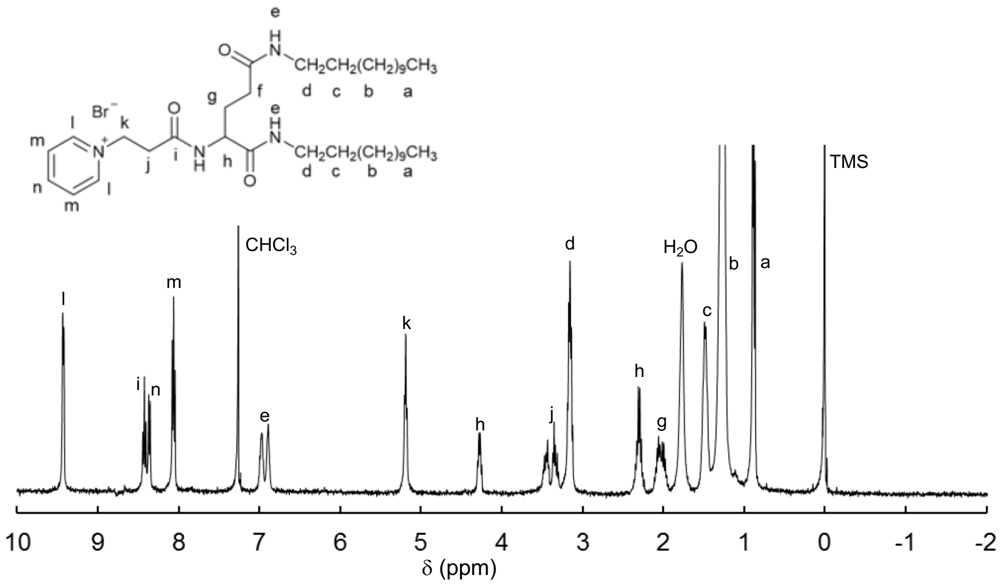

Figure S2 ^{1}H NMR spectrum of *G*-Py+.

Synthesis of S-PEG$_n$-S

Synthesis scheme of S-PEG-S was depicted and the synthesis of each step is described below.

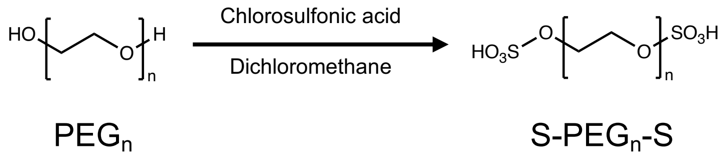

S-PEG$_n$-S

20 g of polyethylene glycol (PEG$_n$) (degree of polymerization (n) = 70, 200, 350) was dissolved in 20 mL of dichloromethane, and the solution was cooled in ice bath. Chlorosulfonic acid (10 equiv.) was added the solution under cooling, and the mixture was stirred at room temperature for 20 h. Thereafter, the solution was concentrated and decanted dropwise into diethyl ether. The precipitate was washed several times with diethyl ether, collected by filtration and dried under vacuum to obtain white powder. ^{1}H NMR spectra of PEG$_{200}$ and S-PEG$_{200}$-S were shown in Figure S3. ^{1}H NMR(CDCl$_3$) : δ 8.10-10.45 (s, 1H, SO_3H), 4.28 (s, 2H, CH_2SO_3H), 3.49–3.66 (m, PEG).

Figure S3

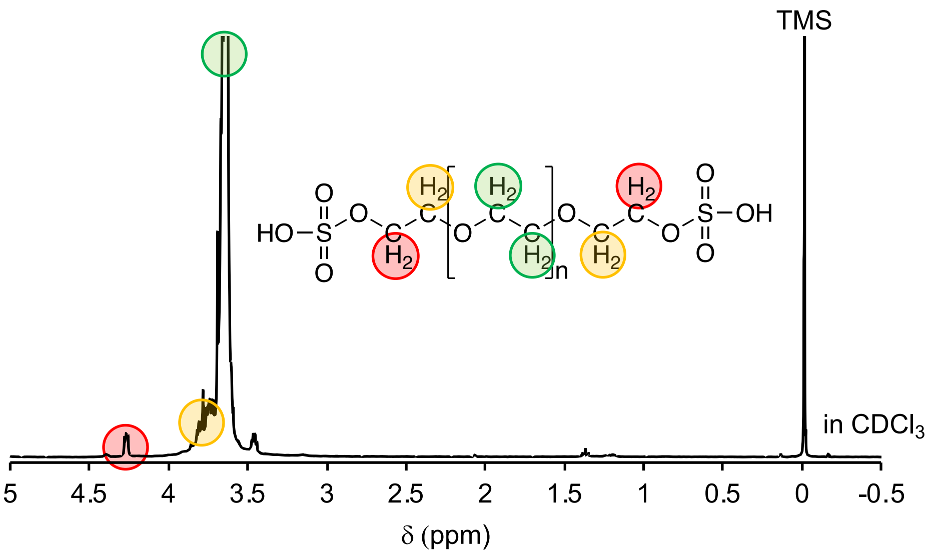

Figure S3 ^{1}H NMR spectrum of S-PEG$_{200}$-S. Solvent: $CDCl_3$.

Figure S4

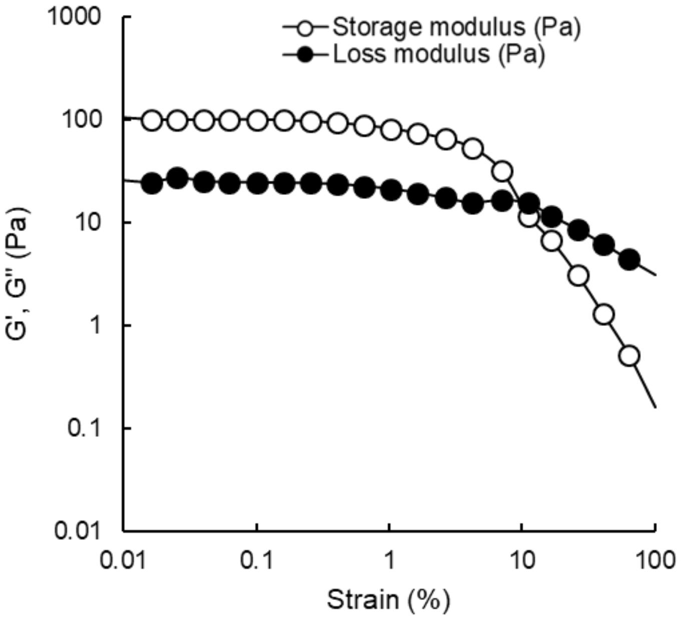

Figure S4 Strain dependence of elastic storage moduli (G′) and viscous loss moduli (G′′) for an aqueous mixture of *G*-Py^{+} and S-PEG$_{n}$-S (10 mM/6 wt%). Temperature: 10 °C; Aging time: 1 h; fFrequency: 1 Hz; Oscillation strain: 0.01 % – 100 %.

Figure S5

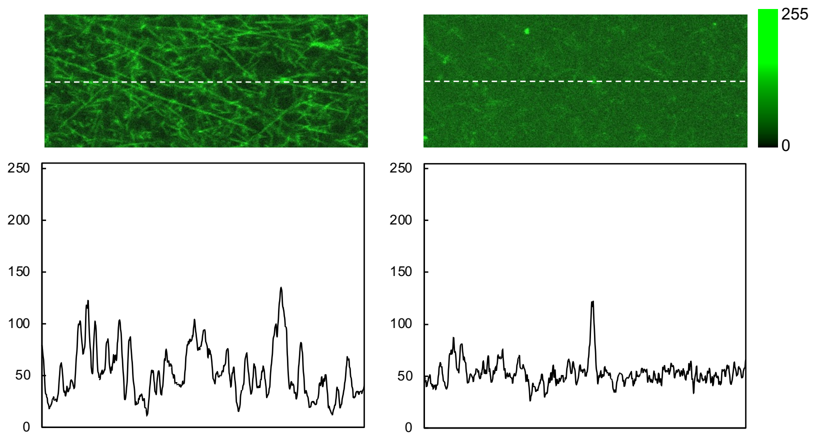

Figure S5 Confocal laser scanning microscopy images and brightness profiles of the dashed line in each figure of G-Py+ (0.5 mM) self-assemblies (left) without and (right) with S-PEG200-S (1 wt%).

Figure S6

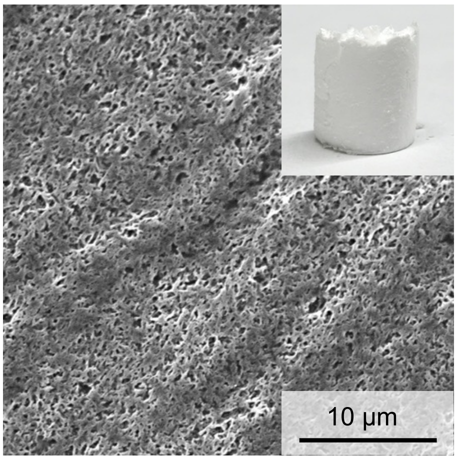

Figure S6 SEM image of *G*-Py$^+$ (10 mM) self-assemblies with PEG_{200} (6 wt%).

Figure S7

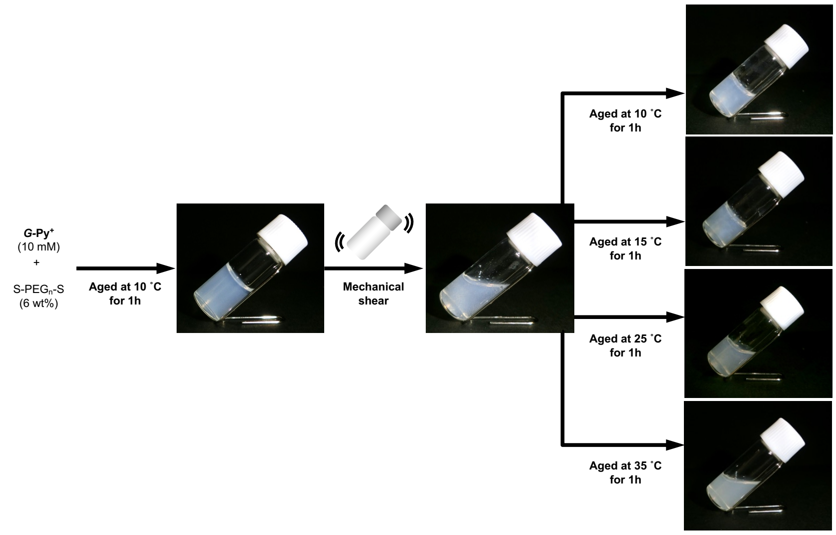

Figure S7 Self-healing properties of the hydrogel formed from an aqueous mixture of G-Py$^+$ and S-PEG_{200}-S at varying temperature. The hydrogel was vortexed to mechanically break it. [G-Py$^+$] = 10 mM; [S-PEG_{200}-S] = 6 wt%.
